# Exploring Perspectives of Parturient Women and the Obstetric Team: Towards an Integrative Obstetric Care Model

**DOI:** 10.3390/healthcare14203421

**Published:** 2026-10-13

**Authors:** Eran Ben-Arye, Orit Gressel, Miri Yahalom, Vered Golan Cohen, Nika Zur, Maayan Lahav Sher, Noah Samuels, Nili Stein, Ofer Lavie, Reuven Kedar

**Affiliations:** 1 Ruth and Bruce Rappaport Faculty of Medicine, Technion-Israel Institute of Technology, Haifa 3525433, Israel; oritgresselraz@gmail.com (O.G.);; 2 Integrative Obstetric Program, Carmel Medical Center, Haifa 3436212, Israel; 3 Department of Obstetrics and Gynecology, Carmel Medical Center, Haifa 3436212, Israel; 4 Center for Integrative Complementary Medicine, Shaare Zedek Medical Center, Faculty of Medicine, Hebrew University of Jerusalem, Jerusalem 9103102, Israel; noahs@szmc.org.il; 5 Department of Community Medicine and Epidemiology, Carmel Medical Center, Haifa 3436212, Israel

**Keywords:** integrative obstetrics, obstetrics, maternity care, parturient women, integrative medicine, acupuncture, complementary medicine, midwifery, mind–body therapies, models of care

## Abstract

**Objectives:** Integrative medicine programs are increasingly being implemented in clinical settings, including gynecology and obstetrics. This study explores an integrative obstetric and maternity care (IObM) model designed for real-life peri-labor settings, comparing perspectives among parturient women to obstetric healthcare professionals (HCPs) regarding operative components of the model. **Methods**: A 26-item questionnaire was prospectively administered to women hospitalized from the 22nd week of gestation (antenatal, early labor, or immediate postpartum) and to obstetric HCPs, primarily obstetricians and nurse-midwives, in three obstetric wards. Four core items addressed key aspects of the IObM model: the source of and indications for referral to IObM treatments, preferred IObM modalities, and willingness to receive care from uncertified IObM providers. **Results**: The two respondent groups (63 parturient women and 60 HCPs; response rate 95 and 100%, respectively) showed similar patterns regarding language, religiosity, spiritual relatedness, prior complementary medicine use (>66%), and perceived effectiveness and risks during pregnancy and labor. Internal consistency within each of the four core items was moderate and acceptable (parturients, α = 0.61–0.69; HCPs, α = 0.62–0.68). HCPs showed greater support than parturients for nurse-midwife referrals (*p* = 0.002) and mind-body trained therapies (*p* = 0.002). Both groups recommended labor-related indications for IObM referral, primarily labor pain, anxiety, and labor progression. Parturients were more supportive of nurse-provided IObM care (*p* = 0.012). **Conclusions**: Exploring expectations of parturient women and HCPs is essential when designing IObM models of care. Four core aspects should be prioritized: source of and indication for the referral to IObM treatments and mapping of preferred IObM modalities and providers. These findings reflect acceptability, expectations, and preferences in a single center with an established integrative service and should not be read as evidence of clinical effectiveness or of successful implementation. Further prospective research should explore implementation of these models in real-life practice, assessing effectiveness, risks, and birth experience.

## 1. Introduction

For many centuries, traditional systems of medicine have been offering a wide range of herbal, manual, relaxation, and movement-related therapies to women during pregnancy and labor [1,2,3]. These modalities have been defined by the World Health Organization as traditional, complementary, and integrative medicine (TCIM) [4], with rates of use for pregnancy-related indications ranging from 20% in the US to 45% and 73% in Germany and Australia, respectively [5,6,7]. The specific TCIM modalities most frequently being used during pregnancy vary between countries, including mind-body medicine (25%, U.S.) [8], touch therapies (43%, China) [9], herbal medicine (38%, U.K.) [10], and spiritual healing (65%, Ethiopia) [11]. Systematic reviews and meta-analyses in the field of TCIM and obstetrics support the safety and effectiveness of many TCIM modalities, including mind-body interventions for anxiety relief during pregnancy [12]; hypnosis [13], acupuncture or acupressure for pain in labor [14]; and acupressure for delayed onset of labor and prolonged labor [15].

As early as 2003, Tiran from the U.K. suggested implementing complementary therapies into midwifery practice [16]. In Australia, Smith and Dahlen followed this call, highlighting the role of acupuncture in changing the maternity service environment [17]. Samuels et al. surveyed nurses and midwives from five Israeli medical centers, reporting that many agreed with fundamental principles of complementary medicine while supporting integration of the parturient woman’s health beliefs and values into maternity care [18]. Encouraged by the recognition and inclusion of integrative oncology services throughout the US and globally [19], integrative medicine practitioners are increasingly developing integrative obstetric and maternity care (IObM) models, emphasizing communication between the obstetric and integrative medicine teams [20]. In a qualitative study conducted in Israel among 22 senior obstetricians and IObM directors from 11 medical centers in which IObM modalities are offered free of charge in the peri-labor setting, a number of barriers and enablers were identified regarding the integrative process [21].

To reduce conceptual ambiguity, the following terms are used consistently throughout this manuscript. Traditional, complementary, and integrative medicine (TCIM) is the umbrella term defined by the World Health Organization [4] and is used here when referring to the international literature and to the modalities themselves. Complementary medicine is used when reporting the wording of the study questionnaire and the responses of participants, since this is the term in common use in Israel and the term that appeared in the Hebrew-language questionnaire. Integrative medicine denotes the evidence-informed provision of these modalities within, and coordinated with, conventional medical care. Integrative obstetric and maternity care (IObM) denotes the specific model examined in the present study: the provision of integrative medicine treatments by certified practitioners within the conventional obstetric setting during the peripartum period, following referral by the obstetric team and with documentation in the patient’s medical file. The terms “alternative” and “complementary/alternative medicine” are used only where they refer to a historical or extra-institutional context and are marked as such.

The goal of the present study was to identify the practical operational aspects of real-life IObM practice, incorporated within the conventional obstetric setting, while acknowledging that any operational model should consider the perspectives and expectations of both the obstetricians, including physicians and midwives/nurses, as well as the women experiencing the parturient journey.

## 2. Methods

### 2.1. Participants and Setting

Between December 2023 and June 2026, the study recruited a convenience sample of parturient women hospitalized in the obstetric wards at the Carmel Medical Center in Haifa, Israel. Eligible participants were women aged ≥18, from the 22nd week of gestation. At the same time, all obstetric-practicing healthcare professionals (HCPs) working in the Department of Gynecology and Obstetrics (residents, specialists, nurses, and midwives) were invited to participate in the study.

Women were recruited from the three obstetric wards of the department: the high-risk antenatal ward, the delivery room, and the postpartum ward. Women in active labor and women whose clinical condition precluded valid informed consent were not approached. Throughout this manuscript, the term parturient women denotes women hospitalized during this peripartum period, as defined above.

Recruitment extended over a prolonged period (December 2023–June 2026) because it coincided with the war in Israel, during which the study center, located in northern Israel, operated for extended intervals under missile threat, with obstetric hospitalization and deliveries conducted at times in protected areas, with reduced elective activity, and with staffing constraints related to reserve duty. Recruitment was therefore intermittent rather than continuous. The structure, staffing, scope and funding of the IObM program itself and the departmental referral pathways remained unchanged throughout the recruitment period, and no organizational or policy change relating to integrative care took place at the study center during this time.

### 2.2. Study Objectives

The primary objective of the study was to explore the recommended indications for HCP referrals to IObM interventions in the peripartum setting, comparing perspectives of parturient women and those of the obstetric HCP team. Secondary study objectives included a comparison of the perspectives of parturient women and HCPs regarding the source of referral to IObM treatments and to identify the leading IObM modalities to be provided in the IObM setting of care.

### 2.3. Study Design

A questionnaire was developed by the authors based on a tool used in an earlier study comparing integrative obstetric models in Israel [21], along with a questionnaire administered to HCPs, which was developed and validated to explore expectations regarding integrative oncology models of care [22,23]. The first questionnaire draft developed for the study was designed for both parturients and HCPs, with some minor modifications in the two versions. The core four questions included in the questionnaire (Supplementary Materials) were identified in former studies as essential aspects to be considered when developing and implementing integrative medicine models in conventional medical settings [21,22,23,24,25,26]. The four “core” questions included the source of referral to IObM treatments; indications for referral; preferred IObM practitioners; and willingness to receive IObM treatment from uncertified IObM practitioners. The authors used the term *complementary medicine,* which is widely used and commonly accepted to define TCIM in Israel. The initial draft of the questionnaire was presented to a focus group of five parturient women who varied in age, obstetric history, education, and past complementary medicine use. Another focus group included 15 HCPs from both obstetrics (physician, midwives/nurses) and IObM services (physicians, acupuncturists, mind-body, and manual therapists), all with IObM training at the study center. Using feedback from the two focus groups, the questionnaire was then revised and sent for reappraisal by a separate group of obstetricians and midwives, followed by a second process of refinement and clarification. The final patient version of the questionnaire consisted of 26 items, including 11 questions about the parturient’s demographics and 15 questions (10 questions with responses on a Likert-like scale, 3 multiple-choice questions, and two open-ended questions) about the parturient’s use of or attitude toward complementary medicine in delivery rooms and during pre- and postpartum care. The HCP version of the questionnaire was similar, except for an additional open-ended question on how to implement the IObM model in the obstetric department and demographic questions about the HCP’s gender, professional background, and previous TCIM training.

Content and face validity were established through a three-stage iterative process: (i) item generation, drawing on instruments previously developed and applied by our group in the IObM and integrative oncology settings [21,22,23] and on a review of the TCIM–obstetrics literature; (ii) appraisal of every item for relevance, clarity, and comprehensiveness by the two focus groups described above, with items rephrased, merged, or deleted by consensus; and (iii) independent reappraisal by a separate panel of obstetricians and midwives who had not participated in the focus groups, followed by a final round of refinement. The revised version was then pilot tested for comprehensibility and completion time among five parturient women and five HCPs, whose responses were not included in the analysis. No exploratory or confirmatory factor analysis was undertaken: the four core questions were designed as a formative operational map of the components required in order to construct an IObM model (including source of referral, indications, modalities, and providers) rather than as a reflective measure of a single underlying trait, and the sample size did not provide an adequate subject-to-item ratio for factor-analytic validation. A full psychometric evaluation, including dimensionality assessment and test–retest reliability, is planned within the multicenter study currently in preparation.

After signing an informed consent form, parturients were asked to complete the study questionnaire, with the assistance of researchers who had undergone training for this purpose. The HCPs responding to the questionnaire were exempt from providing informed consent and asked to complete the questionnaire independently. All questionnaires were completed anonymously, and survey data were entered into a computer database (Microsoft Excel file) for analysis.

### 2.4. Data Analysis

Statistical analysis was performed using IBM SPSS Statistics (version 28.0; (IBM, New York, NY, USA) and SAS (version 9.4; SAS Institute Inc., Cary, NC, USA). Sample size was determined a priori on the basis of the primary study objective, namely the comparison between parturient women and obstetric HCPs of the recommended indications for referral to IObM interventions. Each indication is expressed as the proportion of respondents endorsing it, and comparisons were planned using a two-sided χ^2^ test at α = 0.05. A sample of 60 respondents per group provides 80% power to detect a standardized effect size of 0.5 (Cohen’s h) corresponding, for example, to a difference between 50% and 75% endorsement of a given indication rated on 1–7 Likert-type scales, 80% power to detect a standardized mean difference (Cohen’s d) of 0.5. The achieved sample (63 parturient women and 60 HCPs) corresponds to a minimal detectable standardized effect of 0.51 at 80% power and α = 0.05. The study was not powered for subgroup analyses within the HCP group, nor for the multivariable models. Continuous variables were presented using means and standard deviations or median and interquartile ranges (IQRs), while categorical variables were presented as counts and proportions. Comparisons of demographical and perspectives towards IObM settings of care between parturient women and obstetric HCPs were analyzed using independent t-test or Mann–Whitney for continuous variables and a Chi square test for categorical variables. An ordinal mixed-effects model with cumulative logit link function was used to assess differences in treatment preference scores between HCPs and parturient women and between the 3 IObM modalities (Traditional Chinese Medicine, TCM; touch; and mind-body) adjusted for baseline characteristics and taking into account repeated measures (i.e., multiple treatment ratings per subject). A multivariable model adjusted for baseline characteristics (age, language, country of birth, ethnicity, income, level of religious observance, family status, and previous experience with TCM/touch/mind-body therapies) was applied with the goal of further assessing respondents’ perspectives regarding the three IObM treatment modalities.

Covariates for the multivariable models were selected a priori and not by data-driven stepwise procedures. They comprised (i) baseline characteristics differing between the two study groups at *p* < 0.10 on univariable comparison (age, country of birth, educational level, family status, and ethnicity) and (ii) variables previously reported to be associated with complementary medicine use and attitudes (primary language, level of religious observance, income, and previous personal experience with TCM, touch, or mind–body therapies) [5,18,22].

The study was designed as an exploratory mapping of the operational components of an IObM model. Comparisons were pre-specified within four families corresponding to the four core questions (sources of referral, indications for referral, treatment modalities, and providers), but no formal adjustment for multiplicity was applied, and all *p*-values are reported unadjusted. For orientation, the Bonferroni-corrected threshold within each family is indicated in the Results, and findings with *p*-values close to 0.05 are presented as hypothesis-generating and as requiring confirmation.

Internal consistency was assessed separately for the parturient and HCP versions of the questionnaire, within each of the four core questions (see Supplementary Material S1). The first two core questions (1 and 2) are multiple-response questions, with respondents able to provide more than one answer for each sub-question (item) and the replies thus analyzed as a dichotomous variable (yes/no). Cronbach’s alpha was calculated for each of the sub-questions in questions 1 and 2. Questions 3 and 4 comprise a set of parallel items sharing a common stem and an identical Likert-type response format. Cronbach’s alpha is reported as an index of the homogeneity of the items within a given core question. It is not presented as evidence that the four core questions together constitute a single unidimensional scale. The composite alpha across all four questions is reported for descriptive purposes only: the four domains are conceptually distinct and jointly define rather than reflect the operational IObM model and thus constitute a formative rather than a reflective construct, for which coefficient alpha is not an appropriate index of construct validity [27]. For a newly developed, exploratory instrument, alpha values of 0.60–0.80 were regarded a priori as moderate but acceptable [28].

### 2.5. Ethical Considerations

The study was part of an ongoing prospective study of the IObM program registry protocol, which had been approved by the Ethics Review Board at the Carmel Medical Center in Haifa, Israel (CMC-0114-23).

## 3. Results

### 3.1. Characteristics of the Study Group

The study included 63 parturient women and 60 HCPs, with a response rate of 95% and 100%, respectively. Of the 66 parturient women approached, 63 completed the questionnaire, and all 60 HCPs employed in the department at the time of the survey responded. All of the parturient respondents completed the questionnaire while hospitalized in the antenatal ward (21, 33.3%), in the delivery room (34, 54%), or in the postpartum ward (8, 12.7%), at a median gestational age of 38 weeks and 2 days (IQR: from 36 weeks + 2 days to 40 weeks). The overall sample of HCPs was predominantly female (92%), the majority midwives/nurses (58%) and physicians (38%), with only five HCPs (8.3%) reporting having had any complementary medicine training. Within the HCP group, physicians and midwives/nurses did not differ significantly in their overall support for the IObM model or across the four core domains (all *p* > [0.05]); these comparisons are underpowered and are reported descriptively only. Table 1 shows that there were no significant differences between the study groups with respect to their primary language (with a predominance of Hebrew in both), level of religious observance, self-assessed relatedness to spiritual content, prior complementary medicine use (over 66%), and prior experience with complementary medicine modalities. Compared with the HCP group, parturients were significantly younger (33.6 ± 4.7 vs. 40.5 ± 10.6; *p* < 0.001), Israeli-born (*p* = 0.016), more likely to be married (*p* = 0.018), less likely to have an academic background (*p* < 0.001), and more likely to be Jewish, though this with borderline significance (*p* = 0.05). At the same time, respondents in both groups expressed similar perspectives regarding complementary medicine, with over 98% supporting its overall effectiveness in obstetrics (median of ≥5 on a 1 to 7 Likert scale score). The two groups also shared a belief that complementary medicine was not associated with risks during pregnancy and labor and were highly supportive of including an IObM model of care during labor and obstetric-related hospitalization.

### 3.2. Reliability of the Four Core Questions in the IObM Questionnaire

Internal consistency within each of the four core questions addressing the perceived IObM model of care was moderate and acceptable [27,28], with Cronbach’s alpha values ranging from 0.613 to 0.689 in the parturient group and from 0.624 to 0.681 in the HCP group. The composite value across the four questions, reported for descriptive purposes only, was α = 0.66 in the parturient group and 0.68 in the HCP group.

### 3.3. Source of Referral to IObM Treatment

**Figure 1:**
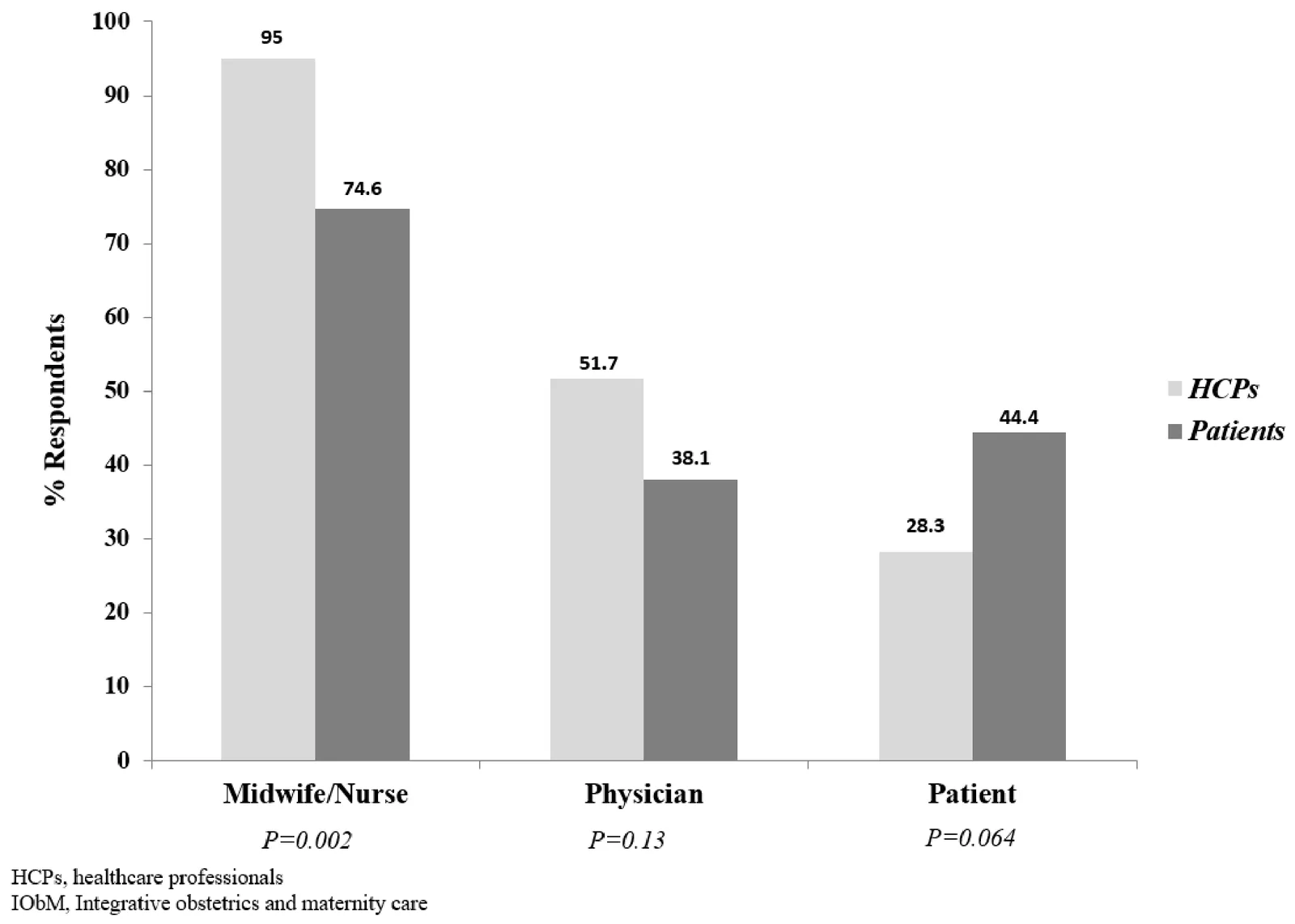
Compared perspectives of OB/GYN HCPs vs. parturients on who should refer to IObM.

Figure 1 illustrates the perspectives of parturients and HCPs vis-à-vis the source of referral to IObM treatments. Compared with parturients, HCPs were more likely to support a nurse/midwife referral (95% vs. 74.6%, *p* = 0.002), while the preference for a physician referral was not significantly different between the two groups (51.7% vs. 38.1%, *p* = 0.13). At the same time, parturients were more likely to prefer self referral over HCP referral during obstetric hospitalization, including labor, though this difference was only marginally significant (*p* = 0.064).

### 3.4. Indications for Referral to IObM Treatments

**Figure 2:**
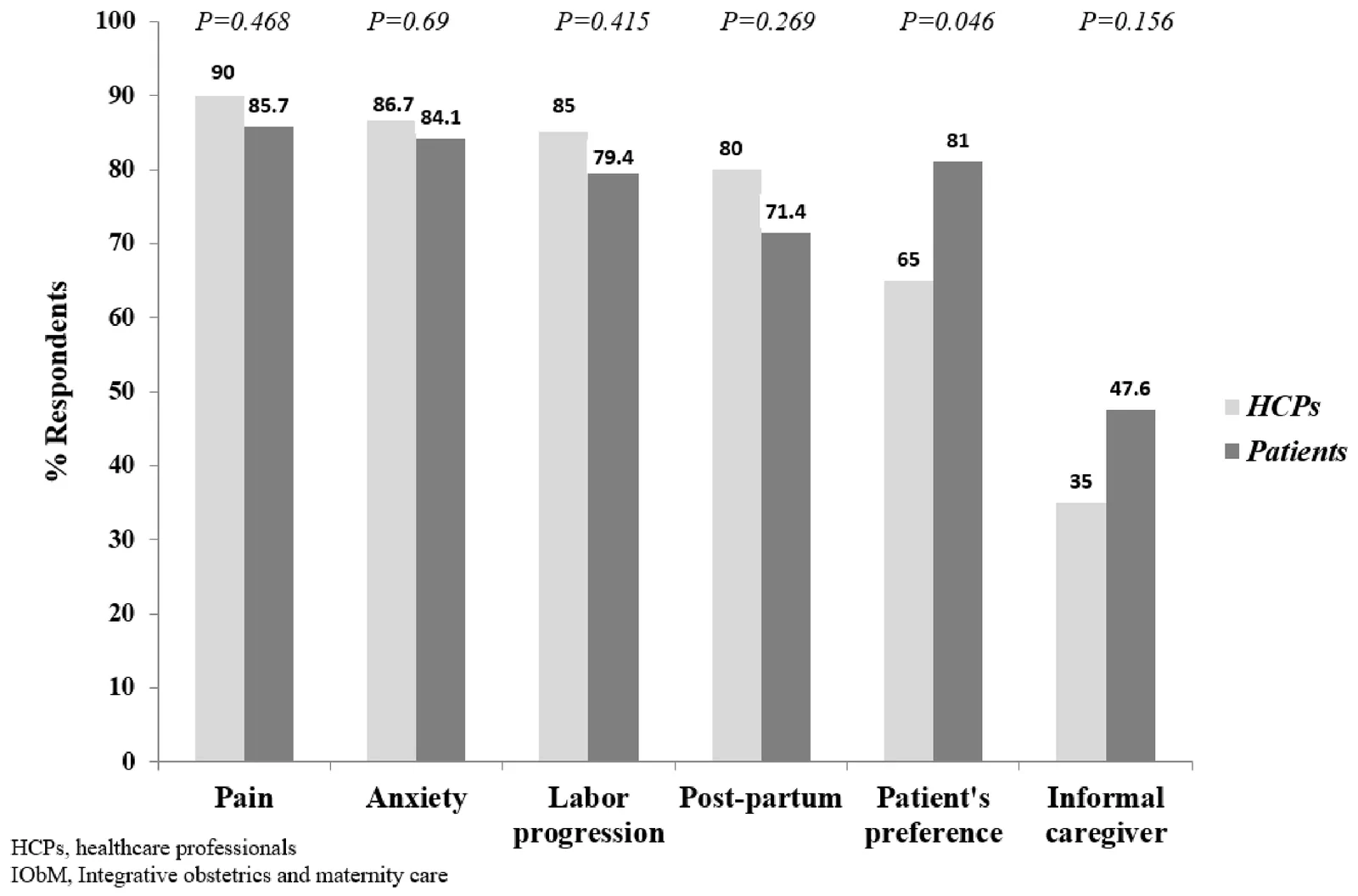
Compared perspectives of OB/GYN HCPs vs. parturients on referral indications to IObM.

Figure 2 indicates that there was broad agreement between parturients and HCPs regarding labor-related indications for referral to IObM treatments, especially with regard to pain, anxiety, and labor progression, as well as postpartum quality of life (QoL)-related concerns during hospitalization. At the same time, 81% of parturients and 65% of HCPs (*p* = 0.046) also supported referral indications that addressed the preference of the parturient woman. Respondents in both groups (a third of HCPs, nearly half of parturients) supported including the concerns of the accompanying caregiver during labor as an indication for the referral. Within the family of comparisons relating to referral indications, the Bonferroni-corrected significance threshold was *p* < [0.05/k]; the between-group difference regarding the parturient woman’s own preference as a referral indication (*p* = 0.046) does not meet this threshold and should therefore be regarded as exploratory.

### 3.5. Preferred IObM Treatment Modalities

**Figure 3:**
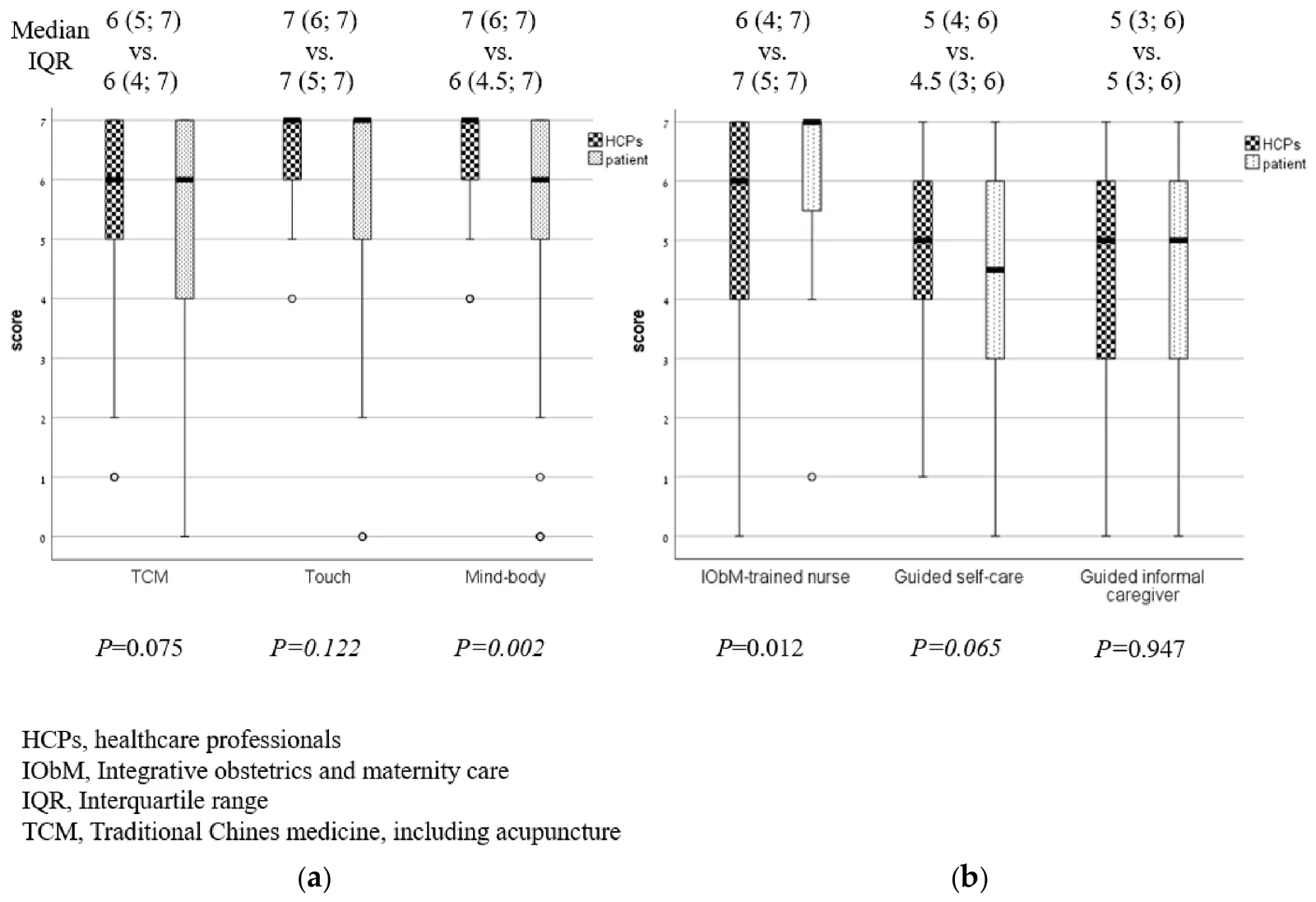
(**a**) HCPs vs. parturients perspectives of preferred IObM modalities; (**b**) HCPs vs. parturients perspectives of preferred provider other than certified IObM practitioners.

Figure 3 presents two aspects of the perceived IObM treatment modalities and providers in the peripartum setting. Figure 3a compares the expectations of HCPs with those of parturient women regarding three IObM treatment modalities: TCM (including acupuncture), touch, and mind-body medicine. Both HCPs and parturient women showed a high level of interest for all three IObM modalities, with median scores ranging from 6 to 7. However, HCPs were significantly more likely to prefer mind-body therapies [median of 7, IQR (6; 7) vs. 6, IQR (4.5–7), *p* = 0.002]. The preference for touch therapies did not differ between groups, and TCM was favored to some extent more among HCPs (borderline significance, *p* = 0.075).

An ordinal mixed-effects model testing interaction between the two groups and the three modalities indicated that HCPs were more likely to support the three IObM treatments than parturient patients [OR = 2.26 95%CI (1.49–3.42), *p* < 0.001]. After adjustment to baseline characteristics, a greater level of interest was found among HCPs when compared to parturients (OR = 2.68 95% CI 1.55- 4.64), *p* < 0.001].

The IObM modality type had an overall significant effect (*p* = 0.01). Pairwise comparisons showed that touch treatments were associated with higher scores when compared to TCM [OR = 2.16 95% CI (1.3–3.54), *p* = 0.003] with no significant differences found between TCM and mind-body or between mind-body and touch modalities. Following adjustment for baseline characteristics, this finding remained significant (OR = 2.32 95%CI 1.39–3.87). Supplement Table S1 presents the results, including OR, 95% CIs, and *p*-values for each variable included in the multivariable model.

### 3.6. Preferred IObM Provider (Other than Certified Practitioner)

Figure 3b compares the perspectives of respondents in the two groups regarding the provision of integrative treatments by non-practitioner nurses, patients, or accompanying caregivers who had been trained and mentored by certified IObM practitioners. The parturient respondents were more likely than HCPs to agree to the option of nurses providing treatments, if trained and mentored by IObM practitioners (*p* = 0.012). Parturient women were also more likely to support self-administered IObM care, if carried under the guidance and supervision of a certified IObM practitioner (borderline significance of *p* = 0.065). Both groups were equally supportive of the accompanying caregiver providing IObM practitioner-guided treatments, with the goal of alleviating QoL-related concerns during labor and hospitalization. Within this family of three comparisons, the Bonferroni-corrected threshold was *p* < 0.017; the difference regarding nurse-provided treatment (*p* = 0.012) meets this threshold, whereas the difference regarding self-administered care (*p* = 0.065) does not.

## 4. Discussion

The present study compared perspectives between parturient women, including those undergoing labor, with obstetric HCPs, with the goal of designing a feasible and effective IObM model of care. A considerable amount of agreement was found between parturients and HCPs regarding the core elements of the IObM operational model. Respondents in both groups agreed that nurse/midwives and physicians should be the main source for referral, based on clinical indications of pain, anxiety, progression of labor, and postpartum QoL-related concerns. They also highlighted the importance of receiving IObM treatments provided by a professional team of trained acupuncturists, touch, and mind-body therapists.

The focus of the study on the referral process to the IObM program, which was designated as the primary study objective, corresponds with previous research on integrative medicine models, including in the IObM setting [21] as well as other clinical venues (e.g., integrative oncology [25]). Indeed, the integrative medicine literature recognizes the HCP-initiated referral as a fundamental element, ensuring feasibility and successful implementation of the integrative model in real-life clinical practice [29]. In the present study, the support for HCP-generated referrals may also be reflective of the significant advocacy shared by both parturients and HCPs for specific referral indications, such as pain and anxiety during labor, rather than non-specific expectations such as a general feeling of wellbeing. In a prior study of senior obstetricians and directors of IObM programs in 11 medical centers across Israel, the tension separating a medicalized and an IObM model was based on clinical referral indications, and a lifestyle-oriented “tender, loving care,” was identified [21]. From a social-medical perspective, the contemporary IObM model explored in the present study emphasizes the need for medicalization of the integrative model, while focusing on referral indications, symptom-based management, and recording of interventions in the patient’s medical file. This *“integrative”* gesture may be viewed as very different from the practice of *“alternative”* and *“complementary”* medicine, where practitioners often provide treatment outside the conventional obstetric setting. The transition from an “alternative” to an “integrative” context of care needs to be further explored, primarily among women in advanced stages of pregnancy, regarding their expectations of the integrative process. This is with the understanding that in the past, the use of acupuncture and other complementary medicine modalities in obstetrics was associated with non-medicalization of birth, women’s autonomy, and the call for natural and “gentle birthing” [30]. Qualitative studies are also needed to explore whether these expectations are relevant to contemporary IObM models of care and whether the paradigm change from “alternative” to “integrative” is indeed supported by parturient women or viewed as being too medicalized.

The present study also highlights distinctive perspectives and disagreements between the parturient and HCP groups. In this regard, parturient women tended to be more supportive than HCPs of self-referral to IObM treatments, although this difference did not reach conventional statistical significance (*p* = 0.064) and should be regarded as a non-confirmed trend. They were, however, significantly more likely to endorse referral indications compatible with their own preferences (81% vs. 65%, *p* = 0.046, unadjusted for multiplicity). The study also underlines the role of the nurse/midwife in the integrative process, for which HCPs emphasize the nurse-generated referral, while parturients expect the nurse to provide the integrative treatment themselves, after having undergone IObM training. This patient-centered view of the IObM model envisions a highly active role for the “conventional” nurse, in both the referral process (supported by 74.6% of parturients) and the practical provision of IObM treatments, together with certified professional IObM practitioners. This “dual practitioner” role for nurse-midwives, offering patients “the best of both worlds”, has been highlighted by the call by Levett et al. for “humanizing childbirth” following maternity acupressure training for HCPs, including midwives and nurses in Somaliland, Africa [31], and in India [32].

Translating this “dual practitioner” role into routine practice raises concrete requirements that the present study did not examine and that we believe should be specified before such a model is implemented. First, training: nurses and midwives providing IObM interventions require a structured, competency-based curriculum limited to a defined and evidence-supported set of techniques (e.g., acupressure at specified points, guided imagery and breathing techniques, and basic touch and massage) rather than to an entire therapeutic system; the maternity acupressure programs described by Levett et al. in Somaliland and India offer a workable template [31,32]. Second, competency assessment: initial certification should be followed by supervised practice, a defined minimum caseload, and periodic re-assessment, with ongoing mentoring by certified IObM practitioners. Third, scope of practice and professional responsibility: institutional protocols must state the indications and contraindications for each technique, the threshold at which the nurse or midwife must consult or refer to a certified practitioner or to the obstetric team, and the manner in which the intervention is documented in the medical record. Fourth, institutional governance: implementation requires the endorsement of nursing and obstetric leadership, clarity regarding medico-legal liability and insurance coverage, an adverse-event reporting pathway, and periodic audit; it must also be reconciled with workload and staffing constraints, since the same nurses and midwives carry primary clinical responsibility for intrapartum care, and an added therapeutic role could otherwise compete with that responsibility. Finally, the boundary between the nurse- or midwife-delivered intervention and that of the certified IObM practitioner needs to be made explicit, so that the model widens access to supportive care without diluting professional standards.

The present study also suggests that parturient women perceive their accompanying caregivers (i.e., spouse, family member, friend, or other) as a participant in the IObM model of care and as someone who can be guided by the IObM practitioner to provide treatments. The multifaceted perspective of the accompanying caregiver, who also needs attention and referral due to QoL-related concerns, while at the same time playing a role as someone who can also provide care, highlights their role as both a partner to the *heroine* giving birth and as a “legitimate” client for an IObM intervention [33]. In a study from Iran, Najafi et al. reported that the presence of a husband, family member, or doula during labor helped parturient women deal more effectively with the labor process [34]. Other studies have suggested that the husband/spouse may experience the pregnancy process with their own QoL-related concerns. This includes feeling excluded from perinatal screening decisions [35], fear of childbirth [36], and postpartum experiences of changing identity, relationships, and future role in the family (e.g., as a father), particularly if this is their first pregnancy [37].

A further finding meriting comment is the support expressed by respondents in both groups for postpartum QoL-related indications for referral. Postpartum quality of life is, however, a multidimensional construct extending well beyond immediate physical recovery and encompassing fatigue and sleep disruption, pain and wound healing, urinary retention, constipation, lactation difficulties, emotional and physical bonding between the mother and the newborn, mood and anxiety, body image, the couple relationship, and sexual well-being. These dimensions are frequently under-recognized in routine postnatal care and ones that women themselves are often reluctant to raise. Recent work has underlined the need to assess these domains using validated, postpartum-specific instruments; Brezeanu et al., for example, recently developed and validated a questionnaire for postpartum quality of sexual life [38]. Future IObM research should specify the targeted postpartum domains a priori and assess them with validated patient-reported outcome measures, which would also allow the scope of supportive and integrative obstetric care to be delineated more precisely and would clarify whether interventions delivered in the peripartum setting influence aspects of postpartum recovery extending beyond the hospitalization itself.

In addition to the study’s findings regarding agreement or divergence in the perspectives of the two groups, a number of insights were reached with respect to the broader conceptual and practical considerations essential to the creation and implementation of the IObM model of care. The transition from “alternative” to “integrative” settings of practice reflects not only a shift in the treatment paradigm but also a reconfiguration of expectations, authority, and roles within obstetric care. Women tend to view IObM as a means for enhancing autonomy, emotional support, and birth experience, whereas HCPs often frame it as a symptom-oriented adjunct option, aligned with medicalized labor management [39]. This gap in perceptions regarding the goal of the IObM program underscores the need for clearer communication and shared decision-making processes. Furthermore, the emerging “dual practitioner” role of nurse/midwives who are positioned between conventional obstetrics and integrative modalities raises questions about training, workload, and professional boundaries. Finally, the study also highlighted the multifaceted role of the accompanying caregiver, perceived simultaneously as a recipient of support and as a potential resource for providing IObM treatments. Collectively, these insights emphasize the complexity of designing an effective IObM model of care, requiring not only the defining of referral pathways and preferred modalities but also addressing cultural expectations, interprofessional collaboration, and the evolving dynamics of childbirth experiences.

Two qualifications should be kept in view when reading the foregoing. The first concerns what the study measures. The present findings describe acceptability, expectations, and preferences, and not clinical effectiveness. That a woman or a clinician endorses a given referral source, indication, modality, or provider indicates what would be considered acceptable and desirable within an IObM model; it says nothing about whether the corresponding intervention relieves labor pain or anxiety, influences the progression of labor, or improves postpartum quality of life, nor about whether such a model can be sustained in routine practice. Evidence on effectiveness must come from the trial literature [12,13,14,15] and from prospective implementation studies, and the two questions should not be conflated.

Also, the uniformly high level of support recorded in both groups requires cautious interpretation. More than two-thirds of respondents in each group reported previous personal use of complementary medicine, over 98% endorsed its effectiveness in obstetrics, and few considered it to carry risk during pregnancy and labor. Such a distribution is unlikely to be representative of parturient women or of obstetric HCPs at large. It may be a product of the recruitment strategy and of the setting: a convenience sample recruited in a department in which an IObM program has been operating for several years and in which integrative treatments are offered free of charge in the peri-labor setting. This may over-represent both women and staff with a pre-existing affinity for integrative care, while those holding reservations may have been less willing to take part. The findings should therefore be read as a description of the expectations held within an integration-receptive environment, which is, in practice, the environment in which such models are first implemented rather than as an estimate of the prevalence of support for IObM in the wider obstetric population.

The present study has a number of methodological limitations that preclude reaching definitive conclusions from the findings. First, the use of a convenience sample may have attracted participants in both groups (parturients and HCPs) who already have an affinity or favorable attitude toward integrative care, potentially influencing their support for the IObM model. Second, the study relied solely on self-reported perceptions rather than behavioral or clinical outcome measures, limiting the ability to determine how stated preferences may have translated into real-world practice. Third, the HCP group was heterogeneous, comprising specialist and resident obstetricians (38%) together with midwives and nurses (58%), whose roles in the peri-labor setting differ substantially. Midwives and nurses are continuously present at the bedside, hold primary responsibility for intrapartum support and comfort measures, and have internationally been the professional group most receptive to complementary therapies [16,17,18], whereas physicians may be involved more episodically and at points of clinical decision-making; residents and specialists may in addition differ in seniority-related attitudes and in previous exposure to integrative care. Pooling these groups may therefore obscure genuine heterogeneity, and the direction of any such difference cannot be inferred from the present data: with 35 midwives/nurses and 23 physicians, the study had adequate power to detect only very large between-profession differences, so that non-significant subgroup comparisons cannot be taken as evidence of equivalence. Profession-stratified sampling, with sample sizes determined for the subgroup comparison itself, is required in order to establish whether the operational preferences described here are shared across the obstetric team; this is of direct practical relevance, since midwives and nurses emerged in the present study both as the preferred source of referral and as preferred providers of treatment. Fourth, the cross-sectional design does not capture the dynamic nature of women’s expectations and needs throughout the different stages of pregnancy and labor, which may shift substantially as clinical circumstances evolve. Respondents were, moreover, questioned at different points of the peripartum journey (antenatal hospitalization, early labor, and the immediate postpartum period), clinical contexts that plausibly shape expectations of supportive care, and the sample size did not permit these strata to be analyzed separately. The prolonged, intermittent recruitment period, which coincided with a period of war in northern Israel, may likewise have influenced attitudes toward anxiety- and distress-related indications. Fifth, the study did not assess organizational factors such as staffing constraints, workflow integration, or institutional readiness, all of which may critically influence the feasibility of implementing the IObM model of care. Sixth and final, cultural and contextual characteristics of the study setting, which took place in a single medical center in Israel that provides a number of integrative medicine programs, including in peri-operative integrative gynecological oncology care [40], may limit the generalizability of the findings for countries where integrative care is less prevalent or else embedded within different sociocultural frameworks. Future research should therefore incorporate longitudinal designs, multi-center sampling, and a mixed-methods approach, including qualitative and narrative-based research, to better capture the complexity of integrating IObM models of care into the diverse obstetric environment. More broadly, the model described here should be regarded as transferable in principle rather than in detail. The four core components (source of referral, indications for referral, preferred modalities, and preferred providers) are likely to be relevant wherever an IObM service is contemplated, and it is this framework, rather than the particular preferences reported by our respondents, that we propose as generalizable. The specific preferences themselves are the product of one clinical, cultural, and regulatory setting, in which integrative services are already established and provided free of charge, and should be re-elicited locally rather than adopted as they stand.

## 5. Conclusions

In conclusion, the present study suggests that exploring expectations of parturient women and their obstetric HCPs may enrich the design of the IObM model of care, based on four “core” implementable aspects of the program, which include identifying the source of referral to IObM treatment; defining the indications for referral; mapping the preferred IObM modalities; and willingness to receive IObM treatment from uncertified IObM practitioners. The model can be also enriched by exploring the parturient’s willingness to be active in both referral and treatment, as well as taking into consideration the role and needs of the accompanying caregiver during the birth journey. Further research should prospectively explore the implementation of the IObM model in real-life obstetric and maternity care. These conclusions describe the acceptability of the model and the design preferences of an integration-receptive, single-center population. They do not establish the clinical effectiveness of IObM interventions, and they require confirmation in multicenter samples that include settings in which integrative services are not yet available.

## Figures and Tables

**Table 1:** Sociodemographic characteristics and perspectives of the study groups.

Characteristic	HCPs N = 60	Parturients N = 63	*p* Values
**Age** mean ± SD	40.5 ± 10.6	33.6 ± 4.7	<0.001
**Gender/Sex** Female	55 (91.7)	63 (100)	*p* = 0.025
**Primary Language** Hebrew Arabic Russian Else	42 (70.0) 10 (16.7) 6 (10.0) 2 (3.3)	51 (81.0) 5 (7.9) 5 (7.9) 2 (3.2)	0.480
**Country of Birth** Israeli born	43 (71.7)	56 (88.9)	0.016
**Familial status** Married	46 (76.7)	58 (92.1)	0.018
**Number of children** Mean ± SD median (IQR)	N = 60 2.2 ± 1.5 2 (1; 3)	N = 62 1.2 ± 1.1 1 (0; 2)	<0.001
**Education** Academic	60 (100)	52 (82.5)	<0.001
**Stated Religion:** Jewish	46 (76.7)	54 (90)	0.050
**Level of Religiosity ** Secular	38 (64.4)	37 (62.7)	0.848
Relatedness to spiritual contents * mean ± SD median (IQR)	3.7 ± 1.9 4 (2,5)	4.1 ± 2.0 4 (2.5, 5.5)	0.237
**Prior CM use ** Yes	42 (70.0)	42 (66.7)	0.691
**CM modalities experienced** TCM Touch-movement Mind-body Dietary/herbal supplements	33 (55.0) 27 (45.0) 10 (16.7) 14 (23.3)	28 (44.4) 32 (50.8) 12 (19.0) 10 (15.9)	0.242 0.520 0.731 0.297
**Overall CM effectiveness in obstetrics ** Yes	60 (100)	62 (98.4)	>0.99
Perceived CM effectiveness * mean ± SD median (IQR)	5.5 ± 1.2 5.5 (5,7)	5.5 ± 1.2 5 (5,7)	0.960
**Overall CM risk in obstetrics ** Yes	5 (8.3)	8 (12.7)	0.431
Perceived CM risk * mean ± SD median (IQR)	1.2 ± 0.78 1 (1,1)	1.2 ± 0.72 1 (1,1)	0.433
Willingness for CM treatment in obstetrics * mean ± SD median	6.0 ± 0.95 6 (5,7)	6.29 ± 1.2 7 (6,7)	0.481

HCPs, healthcare providers. * Data based on a Likert scale scored from 1 (very slightly agree) to 7 (agree very much). CM, complementary medicine; SD, standard deviation; IQR, nterquartile range.

## Data Availability

The de-identified participant-level dataset, the study questionnaire (Hebrew and English versions), and the statistical analysis code that support the findings of this study are available from the corresponding author (E.B.-A., eranben@technion.ac.il) upon reasonable request. The data are not deposited in a public repository because the ethics approval obtained for this study (CMC-0114-23) does not extend to public deposition of participant-level data and because of the small single-center sample. In particular, the healthcare-professional group, which comprises the entire staff of one department, carries a residual risk of re-identification. Requests will be reviewed by the corresponding author and, where participant-level data are requested, forwarded to the Carmel Medical Center Ethics Review Board; access will be granted subject to that approval and to a data-sharing agreement.

## Supplementary Materials

The following supporting information can be downloaded at https://www.mdpi.com/article/10.3390/healthcare14203421/s1: Supplementary Material S1: The 4-core questions included in the study questionnaire.

## References

[1] Michel J., Duarte R.E., Bolton J.L., Huang Y., Caceres A., Veliz M., Soejarto D.D., Mahady G.B. (2007). Medical potential of plants used by the Q’eqchi Maya of Livingston, Guatemala for the treatment of women’s health complaints. J. Ethnopharmacol..

[2] Fok D., Aris I.M., Ho J., Lim S.B., Chua M.C., Pang W.W., Saw S.M., Kwek K., Godfrey K.M., Kramer M.S. (2016). GUSTO Study Group. A Comparison of Practices During the Confinement Period among Chinese, Malay, and Indian Mothers in Singapore. Birth.

[3] Zhang N.H. (1988). Maternity care in China. Midwives Chron..

[4] von Schoen-Angerer T., Wischnewsky M., Jeitler M., Brinkhaus B., Ortiz M., Kessler C.S. (2026). Which Terms for Tra-ditional, Complementary, and Integrative Medicine Would Be the Most Useful in Germany? Results from a Large Online Cross-Sectional Study. Complement. Med. Res..

[5] Johnson P.J., Kozhimannil K.B., Jou J., Ghildayal N., Rockwood T.H. (2016). Complementary and Alternative Medicine Use among Women of Reproductive Age in the United States. Womens Health Issues.

[6] Schürger N., Klein E., Hapfelmeier A., Kiechle M., Paepke D. (2018). Demand for integrative medicine among women in pregnancy and childbed: A German survey on patients’ needs. BMC Complement. Altern. Med..

[7] Skouteris H., Wertheim E.H., Rallis S., Paxton S.J., Kelly L., Milgrom J. (2008). Use of complementary and alternative medicines by a sample of Australian women during pregnancy. Aust. N. Z. J. Obstet. Gynaecol..

[8] Birdee G.S., Kemper K.J., Rothman R., Gardiner P. (2014). Use of complementary and alternative medicine during pregnancy and the postpartum period: An analysis of the National Health Interview Survey. J. Womens Health.

[9] Zeng Y., Zhou Y., Chen P., Luo T., Huang M. (2014). Use of complementary and alternative medicine across the child-birth spectrum in China. Complement. Ther. Med..

[10] Pallivalapila A.R., Stewart D., Shetty A., Pande B., Singh R., McLay J.S. (2015). Use of complementary and alternative medicines during the third trimester. Obstet. Gynecol..

[11] Emiru Y.K., Adamu B.A., Erara M., Chanie T., Gurmu A.E. (2021). Complementary and Alternative Medicine Use in a Pregnant Population, Northwest Ethiopia. Int. J. Reprod. Med..

[12] Marc I., Toureche N., Ernst E., Hodnett E.D., Blanchet C., Dodin S., Njoya M.M. (2011). Mind-body interventions during pregnancy for preventing or treating women’s anxiety. Cochrane Database Syst. Rev..

[13] Madden K., Middleton P., Cyna A.M., Matthewson M., Jones L. (2012). Hypnosis for pain management during labour and childbirth. Cochrane Database Syst. Rev..

[14] Smith C.A., Collins C.T., Levett K.M., Armour M., Dahlen H.G., Tan A.L., Mesgarpour B. (2020). Acupuncture or acu-pressure for pain management during labour. Cochrane Database Syst. Rev..

[15] Mollart L.J., Adam J., Foureur M. (2015). Impact of acupressure on onset of labour and labour duration: A systematic review. Women Birth.

[16] Tiran D. (2003). Implementing complementary therapies into midwifery practice. Complement. Ther. Nurs. Mid-Wifery.

[17] Smith C., Dahlen H. (2009). Caring for the pregnant woman and her baby in a changing maternity service envi-ronment: The role of acupuncture. Acupunct. Med..

[18] Samuels N., Zisk-Rony R.Y., Singer S.R., Dulitzky M., Mankuta D., Shuval J.T., Oberbaum M. (2010). Use of and attitudes toward complementary and alternative medicine among nurse-midwives in Israel. Am. J. Obstet. Gynecol..

[19] Cohen L., Ben-Arye E. (2026). Integrative oncology: Evolution, framework, and future directions. Front. Oncol..

[20] Ben-Arye E., Biron-Shental T., Galil G., Cohen V., Keidar R., Schiff E. (2025). On The Threshold between Birth and Re-lease: An Integrative Obstetrics Intervention. Harefuah.

[21] Ben-Arye E., Biron-Shental T., Rosa W.E., Samuels N., Levy O., Keshet Y., Armon S., Gressel O., Kedar R., Messinger A. (2025). Acupuncture in obstetrics: Delivery room integrative medicine for anxiety and pain. BMJ Support. Palliat. Care.

[22] Ben-Arye E., Massalha E., Bar-Sela G., Silbermann M., Agbarya A., Saad B., Lev E., Schiff E. (2014). Stepping from tradi-tional to integrative medicine: Perspectives of Israeli-Arab patients on complementary medicine’s role in cancer care. Ann. Oncol..

[23] Ben-Arye E., Schiff E., Mutafoglu K., Omran S., Hajjar R., Charalambous H., Dweikat T., Ghrayeb I., Sela G.B., Turker I. (2015). Inte-gration of complementary medicine in supportive cancer care: Survey of health-care providers’ perspectives from 16 countries in the Middle East. Support. Care Cancer.

[24] Ben-Arye E., Schiff E., Zollman C., Heusser P., Mountford P., Frenkel M., Bar-Sela G., Lavie O. (2013). Integrating com-plementary medicine in supportive cancer care models across four continents. Med. Oncol..

[25] Shalom-Sharabi I., Frenkel M., Caspi O., Bar-Sela G., Toledano M., Samuels N., Schiff E., Ben-Arye E. (2018). Integrative Oncology in Supportive Cancer Care in Israel. Integr. Cancer Ther..

[26] Burton W., Salsbury S.A., Goertz C.M. (2024). Healthcare provider perspectives on integrating a comprehensive spine care model in an academic health system: A cross-sectional survey. BMC Health Serv. Res..

[27] Streiner D.L. (2003). Starting at the beginning: An introduction to coefficient alpha and internal consistency. J. Pers. Assess..

[28] Nunnally J.C., Bernstein I.H. (1994). Psychometric Theory.

[29] Bazzell A.F., Madsen L.T., Bandini L., Harrington T., Siman J., Stein C.E., Greenlee H., Fleming J.B., Huffman A. (2026). In-tegrative Oncology Models of Care: Practice Patterns from NCCN Member Institutions. J. Adv. Pract. Oncol..

[30] Hrešanová E. (2018). The Island of Alternatives: Power, Medical Science, and “Gentle Birthing” in Socialist Czecho-slovakia. J. Hist. Med. Allied Sci..

[31] Levett K.M., Salomons E., Jirde I.S., Ahmed R., Isamil E.A. (2026). Humanising childbirth: Evaluation of maternity acu-pressure training for healthcare providers at Edna Adan University and Hospital in Hargeisa, Somaliland, Africa. Women Birth.

[32] Levett K.M., Salomons E., Shenoy P., Kaur I., Fernandez E. (2024). Humanising childbirth—Maternity acupressure training for healthcare providers at the Fernandez Foundation Hospitals, Hyderabad, India. Evaluation of program delivery in one region of India. Women Birth.

[33] Jolliffe R.C., Durrant J.R., Seers H.E., Churchward S.F., Griffiths M., Naidoo M., Ben-Arye E., Polley M.J., Zollman C. (2018). Impact of Penny Brohn UK’s Living Well Course on Informal Caregivers of People with Cancer. J. Altern. Complement. Med..

[34] Fathi Najafi T., Latifnejad Roudsari R., Ebrahimipour H. (2017). The best encouraging persons in labor: A content analysis of Iranian mothers’ experiences of labor support. PLoS ONE.

[35] Cheng E.R., McGough H., Tucker Edmonds B. (2019). Paternal Preferences, Perspectives, and Involvement in Perina-tal Decision Making. Obstet. Gynecol. Surv..

[36] Mortazavi F., Nikbakht R., Mehrabadi M., Shahhosseini Z. (2023). A structural equation model analysis of the rela-tionship between expectant fathers’ fear of COVID-19 and their fear of childbirth: The mediating role of maternal fear of childbirth. Midwifery.

[37] Barclay L., Donovan J., Genovese A. (1996). Men’s experiences during their partner’s first pregnancy: A grounded theory analysis. Aust. J. Adv. Nurs..

[38] Brezeanu D., Brezeanu A.M., Surdu T., Tica V.I. (2025). Development and Validation of the Postpartum Quality of Sexual Life Questionnaire: A Prospective Observational Cohort Study. Cureus.

[39] Prosen M., Krajnc M.T. (2019). Perspectives and experiences of healthcare professionals regarding the medicalisa-tion of pregnancy and childbirth. Women Birth.

[40] Ben-Arye E., Samuels N., Schiff E., Lavie O. (2016). Designing an integrative gynecologic oncology model of support-ive care: Call for a cross-cultural international collaboration. Support. Care Cancer.

