# Supplementary material for: Exploring Perspectives of Parturient Women and the Obstetric Team: Towards an Integrative Obstetric Care Model
Source: Healthcare (Basel). 2026 Oct 13;14(20):3421. doi: 10.3390/healthcare14203421 (PMC13704188; doi:10.3390/healthcare14203421)
Supplement: Supplementary file 1 [file healthcare-14-03421-s001.zip › healthcare-4551105-supplementary.pdf]

Supplementary Material S1: The 4-core questions included in the study questionnaire

1. Who, in your opinion, should refer to complementary medicine treatment in the delivery room and hospital setting?

1. The nurse/midwife
2. The physician
3. The patient, without need for a referral by the healthcare team
4. Other. Please specify:_____

2. For which of the following situations, in your opinion, is it worthwhile to receive complementary medicine treatment in the delivery room and hospital setting?

1. Pain during delivery
2. Anxiety before and during delivery
3. Challenges with progression of labor
4. Distress of the accompanying caregiver
5. Difficulties arising after birth
6. According to the woman's wish
7. Other. Please specify:_______

3. To what extent would you like to be treated by the following practitioners of complementary medicine in the delivery room and hospital setting?

a. A traditional Chinese medicine acupuncturist

Not at all 0 To a very low extent 1 ---- 2 ---- 3 ---- 4 ---- 5 ---- 6 ---- 7 To a very significant extent

b. A touch and movement modalities practitioner (reflexology, Shiatsu, Paula method, Feldenkrais exercise, Qi Gong, etc.)

Not at all 0 To a very low extent 1 ---- 2 ---- 3 ---- 4 ---- 5 ---- 6 ---- 7 To a very significant extent

c. A relaxation and breathing modalities practitioner (guided imagery, relaxation and breathing exercises, etc.)

Not at all 0 To a very low extent 1 ---- 2 ---- 3 ---- 4 ---- 5 ---- 6 ---- 7 To a very significant extent

4. To what extent would you like to be treated with complementary medicine in the delivery room and hospital setting by the following?

a. Midwife/nurse who has received training in complementary medicine

Not at all 0 To a very low extent 1 ---- 2 ---- 3 ---- 4 ---- 5 ---- 6 ---- 7 To a very significant extent

b. Self-treatment under the guidance of a complementary medicine practitioner

Not at all 0 To a very low extent 1 ---- 2 ---- 3 ---- 4 ---- 5 ---- 6 ---- 7 To a very significant extent

c. Treatment by the accompanying caregiver under the guidance of the complementary medicine practitioner

Not at all 0 To a very low extent 1 ---- 2 ---- 3 ---- 4 ---- 5 ---- 6 ---- 7 To a very significant extent
